# Humeral torsional side differences after nonoperative treatment of proximal humerus fractures and humeral shaft fractures: clinical and ultrasonographic assessment

**DOI:** 10.1186/s13018-023-03671-2

**Published:** 2023-03-16

**Authors:** Sam Razaeian, Jan-Niklas Menzel, Dafang Zhang, Christian Krettek, Nael Hawi

**Affiliations:** 1 Department of Trauma Surgery, Hannover Medical School, Carl-Neuberg-Str. 1, 30625 Hannover, Germany; 2 Hannover Medical School, Carl-Neuberg-Str. 1, 30625 Hannover, Germany; 3 Department of Orthopaedic Surgery, Brigham and Women’s Hospital, 75 Francis St, Boston, MA 02115 USA; 4 Hannover Humerus Registry (HHR), Traumastiftung gGmbH, Carl-Neuberg-Str. 1, 30625 Hannover, Germany; 5 Orthopaedic and Surgical Clinic Braunschweig (OCP), Mauernstraße 35, 38100 Braunschweig, Germany

**Keywords:** Ultrasonographic humeral torsion, Humeral head retroversion, Proximal humerus fracture, Nonoperative treatment, Humeral shaft fracture

## Abstract

**Background:**

The purposes of this study were to investigate (1) sonographic humeral torsion (SHT) and side differences (∆SHT), and (2) to determine the relationship between SHT and range of rotational motion (RORM) as well as functional outcome scores of nonoperatively treated proximal humerus fractures (PHF) and humeral shaft fractures (HSF).

**Methods:**

Between October 2020 and July 2021, consecutive patients with radiographically healed nonoperatively treated PHF and HSF were included in this analysis. Subjective perception of torsional side difference, correlation between SHT and RORM, Subjective Shoulder Value as well as absolute and adjusted Constant Score were determined. Degree of humeral torsional side differences were classified as follows: 0°–15°: minor; > 15°–30°: moderate; > 30°: major. Factors including gender, hand dominance, fracture type, and displacement were also assessed in order to investigate any association between these variables and ∆SHT.

**Results:**

Sixty-five patients with nonoperatively treated PHF (*n* = 47) and HSF (*n* = 18) were analyzed. Mean follow-up was 13.2 months (range, 2.1–72.6). The majority (80% (52)) resulted in only minor, 15.4% (10) in moderate, and 4.6% (3) in major torsional side differences. Patients with minor or moderate torsional differences did not perceive any subjective side difference. While RORM correlated fairly to highly with functional outcomes, only very low to low correlation was observed between these measures and SHT and ∆SHT. Gender, fracture displacement, and type of fracture were not related to SHT and ∆SHT. However, significantly greater torsional side differences were observed, when the dominant side was involved (*p* = 0.026).

**Conclusion:**

Nonoperative early functional treatment of proximal humerus and humeral shaft fractures results mainly in only minor humeral torsional side differences. Minor and moderate amounts of torsional side differences might not be perceived by patients.

## Introduction

It is known that the majority of proximal humerus fractures (PHF) and humeral shaft fractures (HSF) can be treated nonoperatively with reasonable functional outcomes [1–5]. Usually, nonoperative treatment of both fracture entities has in common an initial period of immobilization in internal rotation of the extremity followed by early functional exercises.

The question arises whether this procedure, especially in a displaced fracture situation, can result in clinically relevant humeral torsional side differences. To date, there is no study investigating this issue.

The purposes of this study were to investigate (1) sonographic humeral torsion (SHT) and side differences (∆SHT), and (2) to determine the relationship between SHT and range of motion as well as functional outcome scores of nonoperatively treated proximal humerus fractures and humeral shaft fractures.

## Materials and methods

### Patients

This study consists of consecutive cases of PHF and HSF from an observational registry study (Hannover Humerus Registry—HHR). HHR is a prospective, single-center registry study of a supraregional Level 1 trauma center, aiming to investigate the healing process of PHF and HSF. The study is authorized by the local ethical committee of Hannover Medical School (3222–2016) and adheres to the CONSORT guidelines by 2010 [6]. All patients gave written consent for participation.

Patients who routinely followed up between October 2020 and July 2021 were included in this analysis. Inclusion criteria were radiographically healed nonoperatively treated PHF with involvement of the surgical neck and HSF. Exclusion criteria included bilateral fractures, fractures of the collum anatomicum, fractures with involvement of the intertubercular sulcus or loss of osseous integrity between the intertubercular sulcus, and humeral head, radiographic signs of delayed union and non-union, concomitant forearm fractures, and cognitive disorders such as dementia. These criteria were judged by one fellowship-trained senior physician with special focus on upper extremity surgery (N.H.), and one senior resident of orthopedic trauma surgery (S.R.).

**Fig. 1:**
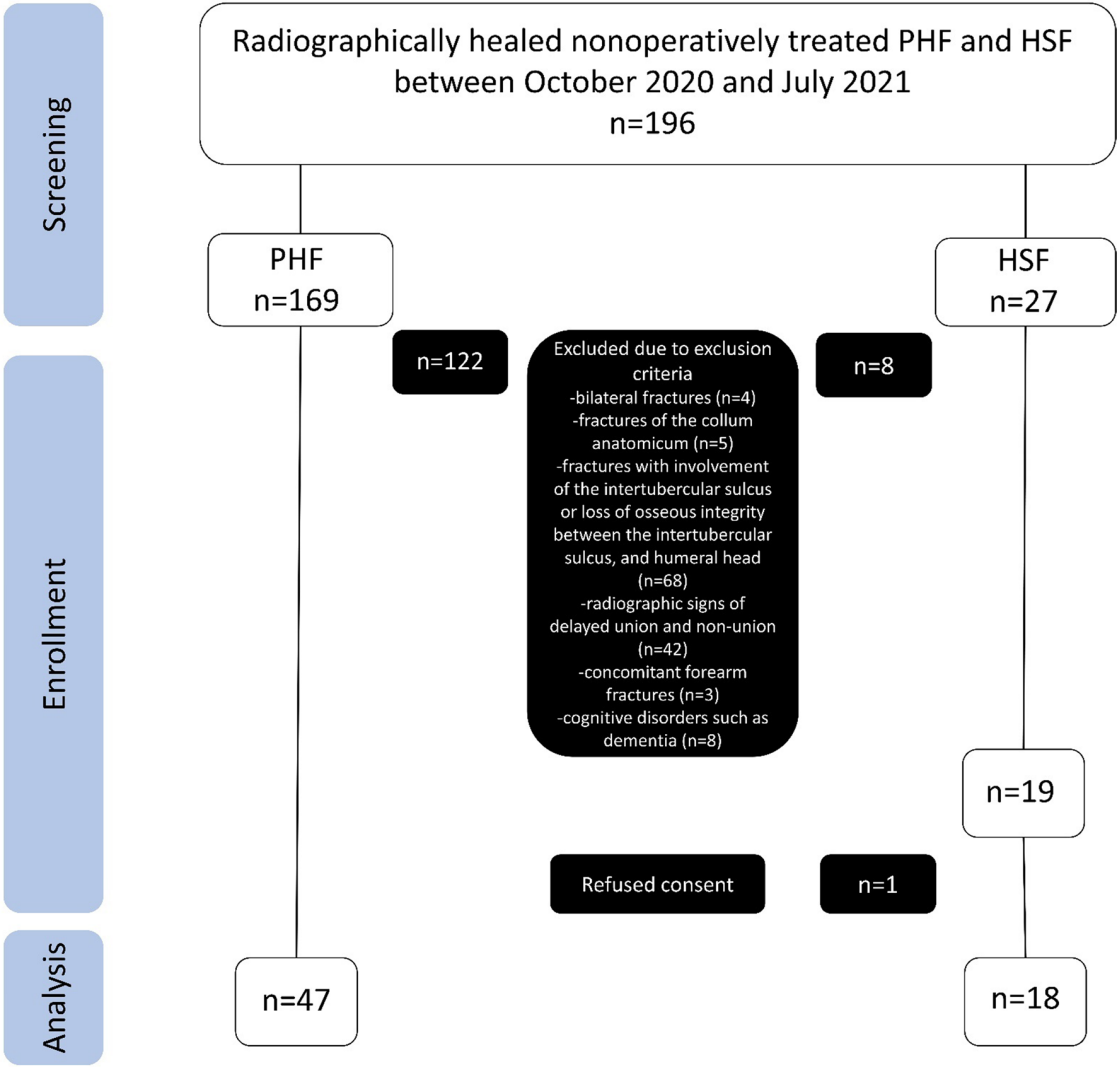
Flow chart. One patient with combined PHF and HSF is categorized as HSF

Figure 1 shows details of inclusion within a flow chart.

PHF were classified according to Neer classification system, and HSF were classified in an abbreviated manner of AO/OTA classification system 2018 by one senior resident of orthopedic trauma surgery trained in classification systems of PHF (S.R.) with the picture archiving and communication software Visage 7.1 (Visage Imaging Inc., San Diego, CA, United States) [7, 8]. In addition, fracture displacement as a dichotomous variable was defined for PHF according to the Neer criteria. In cases of HSF, any radiographic angulation in the coronal or sagittal plane of more than 30°, shortening of more than 3 cm, and ad latus of more than the shaft width was set as displacement criteria.

Humeral torsion was measured by one observer (J.M.) according to the method described by Ito with a 7.5 MHz linear transducer of a conventional ultrasonographic device (Affinity 70G, Koninklijke Philips N.V., 1096 BC Amsterdam, Netherlands) [9]. Degree of humeral torsional side differences were classified as follows: 0°–15°: minor; > 15°–30°: moderate; > 30°: major.

In addition, clinical range of rotational motion (internal rotation and external rotation) in 0° and 90° of shoulder abduction was measured by the same observer. For internal rotation in 0° abduction, the degree of internal rotation as noted by the position of the dorsum of the hand was classified as follows: interscapular region: 95°; 12th vertebral body: 90°; belt line: 85°; lumbosacral transition: 80°; buttock: 75°; and lateral thigh: 70°.

Functional outcome scores including the Subjective Shoulder Value (SSV) and absolute Constant Score (CS) were measured by two independent study nurses. CS was age- and gender-adjusted according to Constant (CSC) and Thomas (CST) [10–12].

Correlation between SHT, ∆SHT and RORM as well as side differences in range of rotational motion (∆RORM) and functional outcome scores was calculated. Moreover, factors including gender, hand dominance, fracture type, and displacement were also assessed in order to investigate any association between these variables and ∆SHT.

### Statistical analyses

Descriptive statistics, including means, standard deviations, and ranges, were calculated. For the analyses, SPSS 26 (IBM, Armonk, New York) and Microsoft Excel 2016 (Microsoft Corporation, Redmond, Washington) were used.

For bivariate analysis of correlation between numeric factors, the correlation coefficient was calculated. Pearson correlation coefficient and Spearman’s rho were used for parametric and nonparametric data, respectively. Correlation strength was classified as follows: very high: > 0.90; high: 0.70–0.89; moderate: 0.50–0.69; fair: 0.30–0.49; low: 0.10–0.29; or very low: 0.10. In order to assess potential factors influencing torsional side differences (∆SHT), Mann–Whitney U test and Kruskal–Wallis test were used as nonparametric tests to compare two and three independent groups, respectively.

## Results

Sixty-five patients with nonoperatively treated PHF (*n* = 47) and HSF (*n* = 18) were analyzed. Mean follow-up was 13.2 months (range, 2.1–72.6) in the total cohort. Mean follow-up was shorter in the group of patients with PHF (8.5 months (range, 2.1–71.6)) than in the group of patients with HSF (25.6 months (range, 2.7–72.6)). One patient with combined PHF and HSF was evaluated 23.9 months after injury. All patients with HSF had worn a sling in the initial immobilization period holding the arm in internal rotation. The majority of patients with PHF (87.2% (41)) had also worn a sling. Only 12.8% (6) of patients had worn an abduction brace due to varus deformity and/or greater tuberosity displacement. Table 1 shows details about patients’ characteristics.

**Table 1:** Patients’ characteristics

	PHF	HSF*
*n* (%)	47 (72.3)	18 (27.7)
Age in years, mean ± SD (range)	59.7 ± 23.5 (11–89)	54 ± 23.7 (11–78)
Patient age ≥ 18, *n* (%)	44 (93.6)	11 (61.1)
Female gender, *n* (%)	37 (78.7)	6 (33.3)
Right side involved, *n* (%)	20 (42.6)	9 (50)
Dominant side involved, *n* (%)	19 (40.4)	8 (44.4)
*Neer classification, n (%)*		
I	38 (80.9)	n.m
II	–	n.m
III	8 (17)	n.m
IV	1 (2.1)	n.m
V	–	n.m
VI	–	n.m
*Abbreviated AO/OTA classification, n (%)*		
A	n.m	13 (72.2)
B	n.m	3 (16.7)
C	n.m	2 (11.1)
*Shaft fracture localization (third), n (%)*		
Proximal	n.m	10 (55.6)
Middle	n.m	6 (33.3)
Distal	n.m	2 (11.1)
Displaced, *n* (%)	9 (19.1)	14 (77.8)

*N.m.* Not measured

*One patient with combined PHF and HSF is categorized as HSF

The majority (80% (52)) resulted in only minor, 15.4% (10) in moderate, and 4.6% (3) in major torsional side differences. A subgroup analysis with exclusion of adolescents and one patient with combined PHF and HSF showed a similar distribution with 75.9% (41) resulting in minor, 18.5% (10) in moderate, and 5.6% (3) in major torsional side differences. While none of those patients with minor or moderate torsional differences perceived any subjective side difference, two of three patients with major side differences reported subjective impairment. One patient with major side difference perceived the difference, but did not report impairment (Table 2).

**Table 2:** Categorized patients’ perception of torsional side difference and their corresponding mean ∆SHT

Categorized patients’ perception of torsional side difference (*n*)	Mean ∆SHT in ° ± SD (range)
No subjective side difference (*n* = 62)	8.1 ± 7.1 (0.1–27.1)
(*n* = 51)	*8.7 ± 7.5 (0.1–27.1)*
Subjective side difference, but without impairment (*n* = 1)	35.3
Subjective side difference with impairment (*n* = 2)	44.9 ± 2 (43.5–46.3)

Italic values show corresponding results of subgroup analysis after exclusion of adolescents and one patient with combined PHF and HSF

While range of rotational motion (RORM) correlated fairly to highly with functional outcomes, only very low to low correlations were observed between SHT and ∆SHT with RORM and functional outcomes (Tables 3, 4, 5).

**Table 3:** Pearson correlation coefficient for correlation analysis between functional outcomes and RORM

	SSV	CS	Adj. CSC	Adj. CST
*Left side*				
Active RORM (0° abd.)	0.75**	0.73**	0.66**	0.76**
Passive RORM (0° abd.)	0.78**	0.77**	0.71**	0.80**
Active RORM (90° abd.)	0.75**	0.77**	0.72**	0.78**
Passive RORM (90° abd.)	0.76**	0.8**	0.79**	0.84**
*Right side*				
Active RORM (0° abd.)	0.61**	0.68**	0.58**	0.67**
Passive RORM (0° abd.)	0.58**	0.64**	0.56**	0.65**
Active RORM (90° abd.)	0.54**	0.6**	0.56**	0.59**
Passive RORM (90° abd.)	0.58**	0.65**	0.64**	0.67**

Bold values indicate significance

*Abd.* Abduction

**p < 0.001

**Table 4:** Pearson correlation coefficient for correlation analysis between SHT, RORM, and functional outcomes

	SSV	CS	Adj. CSC	Adj. CST	Active RORM (0° abd.)	Passive RORM (0° abd.)	Active RORM (90° abd.)	Passive RORM (90° abd.)
*Left side*								
SHT	− 0.13	− 0.11	− 0.03	− 0.12	− 0.21	− 0.18	− 0.16	− 0.19
*Right side*								
SHT	− 0.25*	− 0.13	− 0.09	− 0.17	− 0.24	− 0.26*	− 0.24	− 0.16

Bold values indicate significance

*Abd.* Abduction

*p < 0.05

**Table 5:** Spearman’s correlation coefficient for correlation analysis between sonographic humeral torsional side differences (∆SHT), and side differences in range of rotational motion. (∆RORM)

	Active ∆RORM (0° abd.)	Passive ∆RORM (0° abd.)	Active ∆RORM (90° abd.)	Passive ∆RORM (90° abd.)
∆SHT	0.22	0.28*	0.22	0.18

Bold values indicate significance

*Abd.* Abduction

*p < 0.05

Gender, fracture displacement, and type of fracture were not related to ∆SHT. However, significantly greater torsional side differences were observed when the dominant side was involved (*p* = 0.026) (Table 6).

**Table 6:** Potential factors influencing torsional side differences (∆SHT)

Factors	Mean ∆SHT in ° ± SD	*p* value
*Gender*		
Male (*n* = 22)	7.4 ± 7.3	0.22
Female (*n* = 43)	10.7 ± 11	
*Fracture type*		
PHF (*n* = 47)	10.4 ± 10.6	0.46
HSF (*n* = 18)	7.7 ± 8.1	
*Neer classification*		
I (*n* = 38)	10.7 ± 10.9	0.73
III (*n* = 8)	9.3 ± 10	
IV (*n* = 1)	4.2	
*Abbreviated AO/OTA classification*		
A (*n* = 13)	7.5 ± 9.3	0.36
B (*n* = 3)	6.3 ± 2.7	
C (*n* = 2)	10.8 ± 5.2	
*Fracture displaced*		
Yes (*n* = 23)	8.3 ± 8.9	0.37
No (*n* = 42)	10.4 ± 10.6	
*Shaft fracture localization (third)*		
Proximal (*n* = 10)	9.4 ± 10.2	0.35
Middle (*n* = 6)	4.2 ± 3.2	
Distal (*n* = 2)	9.4 ± 4.4	
*Dominant side involved*		
Yes (*n* = 27)	12 ± 10.5	**0.026**
No (*n* = 38)	7.9 ± 9.4	

Bold values indicate significance

## Discussion

### Principal findings

This is the first study investigating sonographic humeral torsion and torsional side differences of nonoperatively treated proximal humerus and humeral shaft fractures.

The fact that the majority of fractures healed with only minor torsional side differences attenuates hypothetical concerns about initial immobilization in internal rotation followed by early functional treatment such as with Codman’s pendulum exercises. Recently, concerns were raised in cases of surgical neck fractures in regard to commercially available slings holding the humeral shaft in an internally rotated position in relation to the humeral head, potentially displacing the fracture and leading to torsional malunion [13]. In cases of humeral shaft fractures, Sarmiento has previously suggested that malrotation of the fragments can be kept within functional and esthetically reasonable ranges with early active contraction of the flexors and extensors of the elbow joint in the form of co-contraction exercises as part of functional, nonoperative treatment [1, 2]. Sarmiento suggested that the triceps, brachialis, and biceps muscles would undergo a coiling of their fibers when the bone fragments rotate due to the fracture, but that they would recoil as the muscles contract during activity. This was believed to realign the fragments in a parallel direction and correct the malrotation [1, 2].

On the contrary, Fjalestad observed a high prevalence of loss of external shoulder rotation (38%) in a retrospective study of 67 humeral diaphyseal fractures treated with functional bracing. In order to evaluate the cause of this loss, humeral torsion was assessed with computed tomography (CT) in 21 patients (31.3%) [14]. Fracture consolidation in malrotation was seen frequently, and a linear correlation between the clinical loss of external rotation and torsional side differences was observed. However, the study was limited by small sample size and selection bias, and the limit intervals were too wide to predict a statistically significant agreement. Consequently, the authors stated that the investigated patients’ loss of external rotation could not be attributed only to osseous malrotation [14].

Moreover, the question of clinical relevance arises, as we observed in our study that torsional side difference even up to 30° might not be perceived by patients. Interestingly, in our study, only very low to low correlations were observed between humeral torsion and range of rotational motion as well as functional outcomes. The same applies for torsional side differences and side differences in total range of rotational motion. These results suggest that bony torsional alterations up to certain degrees might play a rather minor role for range of rotational motion compared with soft tissue factors. Similar low strengths of correlation between sonographic humeral torsion and range of rotational motion were observed by Yamamoto in a cohort of uninjured, adolescent throwing athletes [15].

We observed significantly greater torsional side differences, when the dominant side was involved. The reason for this finding is speculative. We posit that, similar to the growth period, there might be a physiologic derotational process of the humeral head after a fracture and subsequent nonoperative healing process, which might be more compromised if the dominant side is involved as patients might tend to use the dominant side more intensively for rotational movements such as personal hygiene.

### Limitations

This study has several limitations to consider.

Firstly, this study included only a low number of cases. Furthermore, in particular, the cohort of patients with PHF suffers from a considerable selection bias with a large proportion of exclusion mostly due to fractures with involvement of the intertubercular sulcus or loss of osseous integrity between the intertubercular sulcus, and humeral head as the ultrasonographic measurement method is not usable in these cases.

It is unclear how a larger sample size and longer follow-ups would have influenced the findings significantly, especially subjectively perceived torsional side differences. Therefore, this aspect should be interpreted carefully with respect to propagating any cut-off for clinically significant torsional side differences. The same applies to the wide age distribution including also adolescents (PHF: 6.4%, HSF: 38.9%) as their bone remodeling potential differs significantly compared to adults, even though an exclusion of this group revealed similar distribution of torsional side differences.

Secondly, torsional determinations have been performed at different follow-up periods and only once by a single observer. Physiologic, asymptomatic side differences are known, and therefore, in the absence of a control group and in the absence of absolute values or side differences before and immediately after injury, it remains unclear to what extent these results can actually be interpreted as a physiological result of the fracture self-reducing.

In particular, the actual extent of the torsional side difference immediately after fracture and before any manipulation would have been interesting, but was not able to be quantified in our cohort of patients with healed fractures.

Lastly, ultrasonographic one-time measurements have been performed, when the fracture was defined as healed only on plain radiographs. This led not only to different, and partially low follow-up periods, but left open the question whether bony remodeling processes are actually fully completed. For further studies, this limitation should be addressed either by usage of computertomographic assessment or consistent, predefined minimum follow-up periods.

## Conclusion

Nonoperative early functional treatment of proximal humerus and humeral shaft fractures largely results in only minor degrees of torsional side differences. Minor and moderate amounts of torsional side differences might not be perceived by patients.

## Data Availability

The datasets used and/or analyzed during the current study are available from the corresponding author on reasonable request.
